# Prognostic Implication of Longitudinal Changes in Cardiothoracic Ratio and Aortic Arch Calcification in Hemodialysis Patients

**DOI:** 10.3390/jpm11080788

**Published:** 2021-08-12

**Authors:** Tung-Ling Chung, Yi-Hsueh Liu, Jiun-Chi Huang, Pei-Yu Wu, Hung-Pin Tu, Szu-Chia Chen, Jer-Ming Chang

**Affiliations:** 1 Graduate Institute of Medicine, College of Medicine, Kaohsiung Medical University, Kaohsiung 807, Taiwan; kaoru02323@gmail.com; 2 Division of Nephrology, Kaohsiung Veterans General Hospital, Kaohsiung 813, Taiwan; 3 Graduate Institute of Clinical Medicine, College of Medicine, Kaohsiung Medical University, Kaohsiung 807, Taiwan; Liuboy17@gmail.com; 4 Department of Internal Medicine, Kaohsiung Municipal Siaogang Hospital, Kaohsiung Medical University, Kaohsiung 812, Taiwan; karajan77@gmail.com (J.-C.H.); wpuw17@gmail.com (P.-Y.W.); 5 Division of Cardiology, Department of Internal Medicine, Kaohsiung Medical University Hospital, Kaohsiung Medical University, Kaohsiung 807, Taiwan; 6 Division of Nephrology, Department of Internal Medicine, Kaohsiung Medical University Hospital, Kaohsiung Medical University, Kaohsiung 807, Taiwan; jemich@kmu.edu.tw; 7 Faculty of Medicine, College of Medicine, Kaohsiung Medical University, Kaohsiung 807, Taiwan; 8 Department of Public Health and Environmental Medicine, School of Medicine, College of Medicine, Kaohsiung Medical University, Kaohisung 807, Taiwan; p915013@kmu.edu.tw

**Keywords:** cardiothoracic ratio, aortic arch calcification, hemodialysis, cardiovascular and overall mortality

## Abstract

Patients with end-stage renal disease have a high prevalence of cardiovascular disease. Chest radiography can be used to assess cardiothoracic ratio (CTR) and aortic arch calcification (AoAC). The aims of this longitudinal follow-up study were to investigate factors associated with changes in CTR and AoAC and understand whether these changes are associated with overall and cardiovascular mortality in hemodialysis (HD) patients. We enrolled 260 patients undergoing HD who had at least two available chest X-rays from 2008 to 2015. CTR and AoAC were assessed in each patient using measurements from baseline and annual chest X-rays. The CTR increased from 49.05% to 51.86% and the AoAC score increased from 3.84 to 9.73 over 7 years. The estimated slopes were 0.24 (*p* < 0.0001) for CTR and 0.08 (*p* = 0.0441) for AoAC. Increased AoAC, older age, female sex, coronary artery disease, and decreased albumin were associated with an increase in CTR, and older age, cerebrovascular disease, decreased albumin, increased Kt/V, and the use of antiplatelet agents were associated with an increase in AoAC. During follow-up, 136 of the 260 (52.3%) patients died, of whom 72 died due to cardiovascular causes. The change in CTR was greater in those who died (*p* = 0.0125) than in those who survived. The AoAC score was also higher in those who died than in those who survived, although there was no significant difference in the change in AoAC between the two groups (*p* = 0.8035). CTR and AoAC increased significantly over time in the HD patients in this longitudinal follow-up study, and the change in CTR was greater in those who died than in those who survived. Chest radiography is a simple and useful tool to assess the progression of CTR and AoAC as a prognostic marker.

## 1. Introduction

Cardiovascular disease is the leading cause of mortality in patients with end-stage renal disease (ESRD), accounting for 43% of all deaths [1]. Compared to the general population in whom coronary heart disease is the leading cause of cardiac death, cardiomyopathy is the most common cardiovascular-related cause of death in dialysis patients [1]. As many as 75% of dialysis patients have been reported to have left ventricular hypertrophy (LVH) [2], which is characterized by an increased cardiothoracic ratio (CTR) on chest radiograph. A 4-year cohort study suggested an association between a high baseline CTR and increased overall mortality and cardiovascular events [3].

The pathogenesis of cardiovascular disease in patients with chronic kidney disease (CKD) is a multifactorial process. In addition to traditional cardiovascular risk factors, mineral metabolism abnormalities, lower levels of circulating and locally produced calcification inhibitors, and the use of calcium-based phosphorus binders and vitamin D have also been shown to lead to the development of vascular calcification [4]. Several studies have demonstrated that aortic arch calcification (AoAC) can be used to predict cardiovascular events and overall mortality in dialysis patients [5,6,7]. Clinically, vascular calcification can be detected using various imaging modalities, including plain radiography, computed tomography (CT), sonography, magnetic resonance imaging, and 18F-fluoride positron emission tomography [8]. Of these modalities, chest radiography is non-invasive and widely available, and it can be used to evaluate both CTR and AoAC.

Despite the documented associations of CTR and AoAC with overall and cardiovascular mortality in hemodialysis (HD) patients, the significance of their changes over time and their clinical application still remain unclear. Therefore, the aims of this study were to investigate factors associated with changes in CTR and AoAC and explore whether these changes are associated with overall and cardiovascular mortality in HD patients.

## 2. Study Patients and Methods

### 2.1. Study Patients and Design

This longitudinal follow-up study was performed at a dialysis clinic in Kaohsiung Medical University Hospital (KMUH) in Taiwan, and the methods were conducted following the relevant guidelines. The Institutional Review Board of KMUH approved the study protocol. All of the study patients provided written informed consent before being enrolled into this study.

Patients were included if they (1) were >20 years of age, (2) had been receiving maintenance HD therapy three times a week for ≥3 months, and (3) had been prescribed with anti-hypertensive or oral hypoglycemic agents for ≥1 month. Patients with only one chest X-ray measurement during the follow-up period were excluded (n = 63). Patients who had been transferred to another hospital (n = 51) and those who had received a renal transplantation (n = 4) were also excluded. Finally, a total of 260 patients were enrolled from December 2008 to December 2015. For the patients who started HD during or before 2008, their baseline data were defined as those recorded in 2008; for the patients who entered the study from 2009 to 2015, their baseline data were considered to be those recorded in the year of entry.

### 2.2. Evaluation of CTR and AoAC by Chest X-ray

Chest plain film X-rays of the enrolled patients were reviewed by one experienced radiologist who was blinded to the patients’ clinical data. CTR was calculated by dividing the transverse cardiac diameter by the transverse thoracic diameter as measured by chest X-ray. AoAC was measured using the scale developed by Ogawa et al. [9], in which the aortic arch as visualized on chest X-ray is divided into 16 sections by circumference, and the number of calcified sections was counted. Chest X-ray measurements were done for each patient every December. The CTR was defined as the ratio of a transverse diameter of the cardiac shadow to the transverse diameter of the chest on chest X-ray, with cardiomegaly defined as CTR > 50% [10]. Besides, in this study, we used the median score of AoAC to classify abnormal values of AoAC.

### 2.3. Laboratory, Medica, and Demographic Data

Data including age, sex, smoking history (ever vs. never), and comorbidities were obtained from patient interviews and a review of medical records. Fasting blood samples were obtained ≤1 month of enrollment into the study and processed using an autoanalyzer (COBAS Integra 400, Roche Diagnostics GmbH, D-68298, Mannheim, Germany). Dialysis efficiency was assessed according to Kt/V, which was determined using the Daugirdas formula [11]. The ultrafiltration rate was calculated as the volume of fluid removed per second of HD adjusted for the patients’ body weight before HD. In addition, data on prescribed medications, including angiotensin-converting enzyme inhibitors, angiotensin II receptor blockers, antiplatelet agents, and HMG-CoA reductase inhibitors (statins) during the study period, were obtained from the patients’ medical records.

### 2.4. Definitions of Overall and Cardiovascular Mortality

Data on mortality were obtained from the medical records of the patients. The causes of death (overall and cardiovascular) were confirmed by two cardiologists, with any disagreements being resolved through consensus with a third cardiologist. The patients were followed until death or March 2021, whichever occurred first.

### 2.5. Reproducibility

Reproducibility of AoAC measurements was assessed in 30 randomly selected patients by one trained medical doctor and one experienced radiologist. The mean percentage error was calculated by dividing the absolute difference by the average of two observations.

### 2.6. Statistical Analysis

Data were expressed as percentages or mean ± standard deviation. Characteristics of the study patients for the continuous and categorical variables were analyzed by *t*-test/Wilcoxon rank sum test, and the chi-squared test/Fisher exact test, as appropriate, for comparisons between groups. A repeated-measures generalized model analysis was used to evaluate yearly changes in CTR and AoAC. This approach treated each CTR and AoAC measure from each participant as a separate observation and was adjusted for within-participant correlations. Subjects were treated as random effects so the analysis was adjusted to each individual’s own CTR and AoAC levels. An exchangeable working correlation structure was accounted for within-patient correlation. This model also explored the significance of risk factors at individual yearly measures for the CTR and AoAC change in death and non-death groups separately as well as between cardiovascular death and non-death groups. Potential confounding factors were also included in the analysis model as covariates, which included age, sex, smoking history, diabetes mellitus, hypertension, coronary artery disease, cerebrovascular disease, duration of HD, albumin, total cholesterol, triglyceride, fasting glucose, glycated hemoglobin (HbA_1c_), uric acid, calcium–phosphorus product, Kt/V, and medications use. Statistical significance was set at *p* < 0.05. The Kaplan–Meier method was used to plot survival curves for overall and cardiovascular survival. Statistical analysis was performed using the SAS statistical package version 9.4 (SAS Institutes, Cary, NC, USA).

## 3. Results

A total of 260 HD patients were included. The mean age was 57.3 ± 12.4 years, with 127 men and 136 women. The median CTR and AoAC scores were 50% and 3, respectively. The mean percent error for AoAC measurements was 12.3 ± 12.3%.

### 3.1. Comparison of the Clinical Characteristics among These Study Groups

A comparison of the clinical characteristics among these study groups is shown in Table 1. Compared to patients with CTR ≤ 50%, patients with CTR > 50% have higher CTR, higher AoAC, older age, more female, lower smoking history, shorter duration of HD, lower albumin, and higher fasting glucose. Besides, compared to patients with AoAC ≤ 3, patients with AoAC > 3 have higher CTR, higher AoAC, older age, higher prevalence of coronary artery disease, higher prevalence of cerebrovascular disease, lower albumin, higher total calcium, and higher prevalence of antiplatelet agent use.

### 3.2. Time Course of Mean Change in CTR or AoAC from Baseline (0 Year) to 7 Years

**Figure 1:**
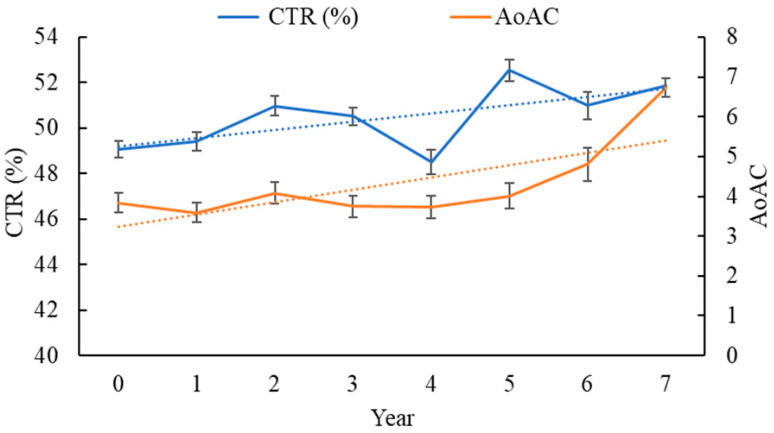
Mean CTR (blue) and AoAC (red) at each follow-up year in study patients (n = 260).

Figure 1 illustrates mean CTR (blue) and AoAC (red) at each follow-up year in the study patients. The CTR (unadjusted β: 0.35, SE: 0.06, *p* < 0.0001) and AoAC (unadjusted β: 0.19, SE: 0.04, *p* < 0.0001) increased yearly.

Table 2 shows the time course of mean change in CTR or AoAC from baseline (0 year) to 7 years. In terms of CTR changes in HD patients, the CTR increased from 49.05% to 51.86% and AoAC score increased from 3.84 to 6.73 over 7 years. When adjusting by all potential confounders in the repeated-measures generalized model, including age, sex, smoking history, diabetes mellitus, hypertension, coronary artery disease, cerebrovascular disease, duration of HD, albumin, total cholesterol, triglyceride, fasting glucose, HbA_1c_, uric acid, calcium–phosphorous product, Kt/V, ultrafiltration rate, and medications use, using the estimated slopes by a repeated-measures generalized model showed 0.24 (95% CI, 0.13 to 0.35; *p* < 0.0001) for CTR and 0.08 (95% CI, 0.002 to 0.16; *p* = 0.0441) for AoAC.

### 3.3. Determinants of CTR and AoAC Yearly Change in Study Patients

Table 3 shows the main effects of the variables on CTR or AoAC change over year in HD patients. In the multivariable analysis, using the repeated-measures generalized model, the main effects of the variables on CTR change in HD patients are increased AoAC (coefficient: 0.11; *p* = 0.0183), age > 60 years old (coefficient: 1.55; *p* = 0.0019), female (vs. male; coefficient: −3.51; *p* < 0.0001), coronary artery disease (coefficient: 1.88; *p* = 0.0038), and decreased albumin (coefficient: −1.86; *p* = 0.0006) were significantly associated with an increase in CTR. As for AoAC, the main effects of the variables on AoAC change in HD patients are age > 60 years old (coefficient: 1.13; *p* = 0.0001), cerebrovascular disease (coefficient: 1.79; *p* = 0.0089), decreased albumin (coefficient: −1.17; *p* < 0.0001), increased Kt/V (coefficient: 1.60; *p* < 0.0001), and antiplatelet agent use (coefficient: 1.01; *p* = 0.0262) were significantly associated with an increase in AoAC.

### 3.4. Risk of Overall Mortality

During the follow-up period, 136 deaths were recorded among these 260 patients (52.3%), including cardiovascular deaths (n = 72), malignancy (n = 14), infectious disease (n = 33), gastrointestinal bleeding (n = 7), and others (n = 10).

A comparison of the clinical characteristics between patients with or without death is shown in Table 4. Compared to patients with non-death, patients with death have higher baseline CTR, higher baseline AoAC, older age, higher smoking history, higher prevalence of diabetes mellitus, higher prevalence of coronary artery disease, higher prevalence of cerebrovascular disease, shorter duration of HD, lower albumin, higher fasting glucose, and higher percentage of antiplatelet agent use.

### 3.5. Time Course of Mean Change in CTR or AoAC from Baseline (0 Year) to 7 Years in Death and Non-Death Groups

**Figure 2:**
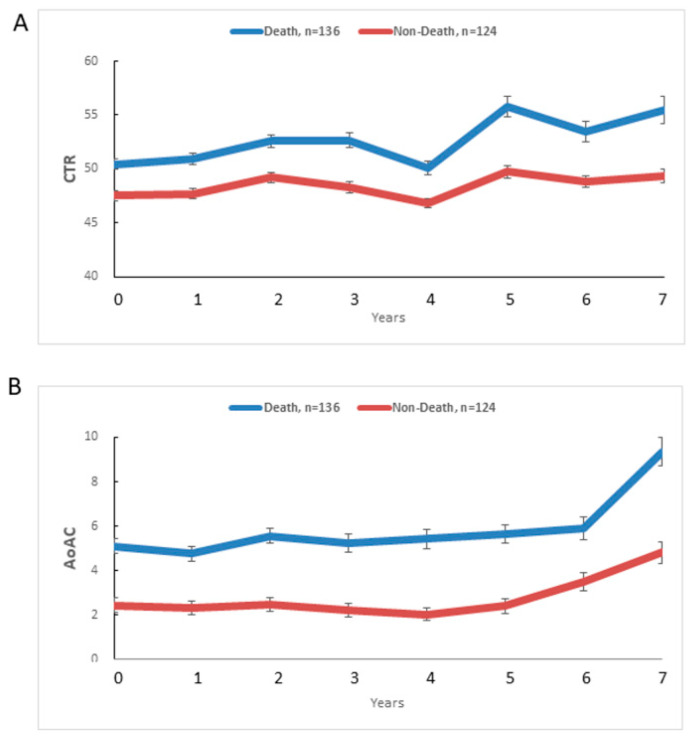
Mean CTR (**A**) and AoAC (**B**) at each follow-up year for death (blue, n = 136) and non-death (red, n = 124) patients.

Figure 2 illustrates mean CTR (A) and AoAC (B) at each follow-up year for death (blue, n = 136) and non-death (red, n = 124) patients. The CTR (unadjusted β: 0.60, SE: 0.11, *p* < 0.0001) increased yearly in the death group, and significantly more than in the non-death group (unadjusted β: 0.21, SE: 0.06, *p* = 0.0002). The AoAC (unadjusted β: 0.23, SE: 0.06, *p* = 0.0002) increased yearly in the death group, and more than in the non-death group (unadjusted β: 0.18, SE: 0.05, *p* = 0.0002), but without achieving significance (*p* = 0.5006).

Table 5 shows the time course of mean change in CTR or AoAC from baseline (0 year) to 7 year in death and non-death groups. In terms of CTR changes, the CTR increased from 50.43% to 55.46% over 7 years in the death group, and from 47.54% to 49.34% in the non-death group. When adjusting by all potential confounders in the repeated-measures generalized model, including age > 60 years old, smoking history, diabetes mellitus, coronary artery disease, cerebrovascular disease, duration of HD, albumin, fasting glucose, and antiplatelet agent use (significant variables in Table 4), using the estimated slopes by a repeated-measures generalized model, the adjusted difference between the death and non-death groups showed 0.30 (95% CI, 0.06 to 0.53; *p* = 0.0125) for CTR.

As for AoAC changes, the AoAC increased from 5.10 to 9.34 over 7 years in the death group, and from 2.45 to 4.81 in the non-death group. The adjusted difference of AoAC in the death group was significantly higher than that in the non-death group at each year (from 0 year to 7 years, *p* ≤ 0.0156), but the yearly change in AoAC between the death and the non-death groups did not achieve significance (*p* = 0.8035). We further performed the time course of logarithmic transformation change in AoAC from baseline (0 year) to 7 years in the death and non-death groups and found similar results.

### 3.6. Risk of Cardiovascular Mortality

The 72 cardiovascular deaths documented during the follow-up included heart failure (n = 30), myocardial infarction (n = 10), ventricular fibrillation (n = 22), and hemorrhagic stroke (n = 10).

### 3.7. Time Course of Mean Change in CTR or AoAC from Baseline (0 Year) to 7 Years in Cardiovascular Death and Non-Death Groups

**Figure 3:**
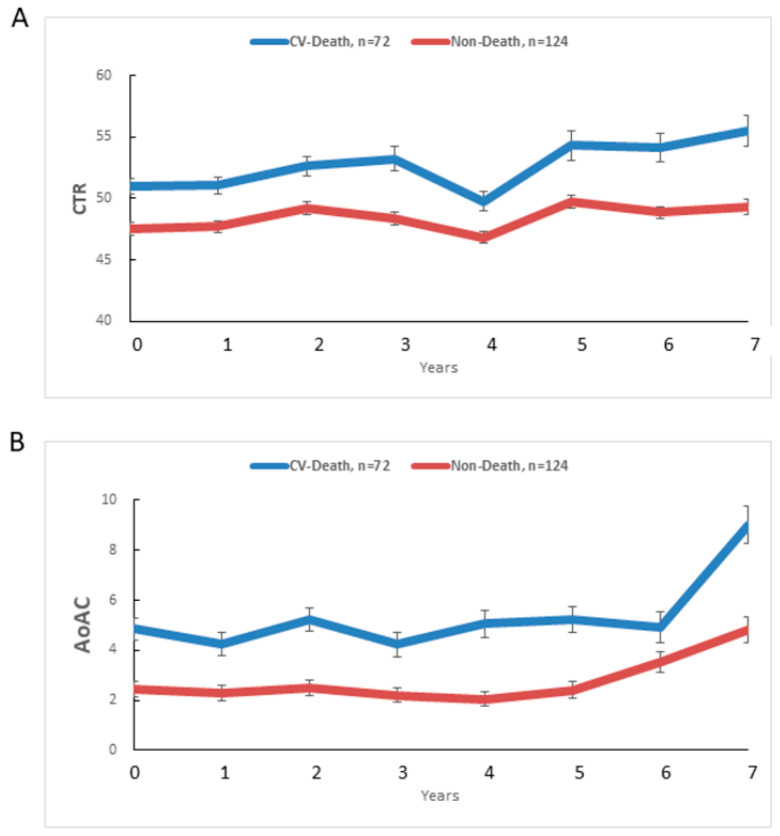
Mean CTR (**A**) and AoAC (**B**) at each follow-up year for cardiovascular death (blue, n = 72) and non-death (red, n = 124) patients.

Figure 3 illustrates mean CTR (A) and AoAC (B) at each follow-up year for cardiovascular death (blue, n = 72) and non-death (red, n = 124) patients. The CTR (unadjusted β: 0.50, SE: 0.14, *p* = 0.0003) increased yearly in the cardiovascular death group, and significantly more than in the non-death group (unadjusted β: 0.21, SE: 0.06, *p* = 0.0002). The AoAC (unadjusted β: 0.17, SE: 0.07, *p* = 0.0185) increased yearly in the cardiovascular death group, and more than in the non-death group (unadjusted β: 0.18, SE: 0.05, *p* = 0.0002), but without achieving significance (*p* = 0.9103).

Table 6 shows the time course of mean change in CTR or AoAC from baseline (0 year) to 7 years in cardiovascular death and non-death groups. In terms of CTR changes, the CTR increased from 50.98% to 55.49% over 7 years in the cardiovascular death group, and from 47.54% to 49.34% in the non-death group. The adjusted difference of CTR in the cardiovascular death group was significantly higher than that in the non-death group at 0, 1, 3, 6, and 7 years, but the yearly change in CTR between cardiovascular death and non-death groups did not achieve significance (*p* = 0.0917).

As for AoAC changes, the AoAC increased from 4.86 to 9.00 over 7 years in the cardiovascular death group, and from 2.45 to 4.81 in the non-death group. The adjusted difference of AoAC in the cardiovascular death group was significantly higher than that in the non-death group at 0, 2, 4, 5, and 7 years, but the yearly change in AoAC between cardiovascular death and non-death groups was not achieving significance (*p* = 0.7461). We further performed the time course of logarithmic transformation change in AoAC from baseline (0 year) to 7 years in cardiovascular death and non-death groups and found similar results.

### 3.8. Correlation between Baseline CTR and Baseline AoAC with Overall Mortality

Table 7 shows the association between baseline CTR and baseline AoAC with overall mortality. After multivariable analysis, baseline CTR (per 1%; odds ratio [OR] = 1.09; 95% confidence interval [CI] = 1.02 to 1.17; *p* = 0.0133), and baseline AoAC (per 1 score; OR = 1.23; 95% CI = 1.11 to 1.37; *p* < 0.0001) were significantly associated with increased overall mortality.

**Figure 4:**
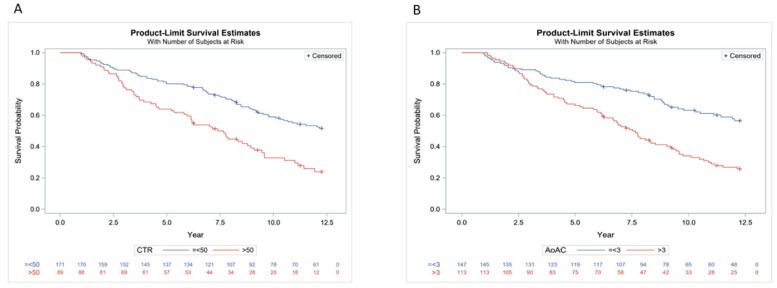
Kaplan–Meier analysis of overall mortality according to (**A**) CTR > 50% or ≤50%, and (**B**) AoAC > 3 or ≤3 in study patients (all log-rank *p* < 0.0001).

**Figure 5:**
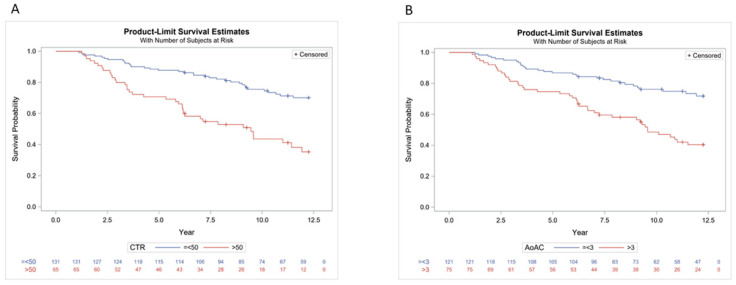
Kaplan–Meier analysis of CV mortality according to (**A**) CTR > 50% or ≤50%, and (**B**) AoAC > 3 or ≤3 in study patients (all log-rank *p* < 0.0001).

We further performed Kaplan–Meier survival curve for overall and cardiovascular mortality. Figure 4 illustrates the Kaplan–Meier curves of overall survival (log-rank *p* < 0.0001) according to (A) CTR > 50% or ≤ 50%, and (B) AoAC > 3 or ≤3 in study patients. The CTR > 50% group had worse overall survival than the ≤ 50% group. The AoAC > 3 group had worse overall survival than the AoAC ≤ 3 group. Figure 5 illustrates the Kaplan–Meier curves of cardiovascular survival (log-rank *p* < 0.0001) according to (A) CTR > 50% or ≤50%, and (B) AoAC > 3 or ≤3 in study patients. The CTR > 50% group had worse cardiovascular survival than the ≤50% group. The AoAC > 3 group had worse cardiovascular survival than the AoAC ≤ 3 group.

### 3.9. Correlation between ΔCTR and ΔAoAC with Overall Mortality

Table 8 shows the association between ΔCTR and ΔAoAC with overall mortality. ΔCTR or ΔAoAC are defined as last year CTR or AoAC minus baseline CTR or AoAC over time. After multivariable analysis, ΔCTR (per 1%; OR = 1.13; 95% CI = 1.06 to 1.21; *p* = 0.0003) was significantly associated with increased overall mortality. However, ΔAoAC (per 1 score; OR = 0.97; 95% CI = 0.88 to 1.08; *p* = 0.6084) was not.

## 4. Discussion

In this cohort study, we demonstrated that CTR increased significantly over time in patients undergoing HD, and that the change in CTR was greater in those who died than in those who survived. In addition, AoAC significantly increased over time, and the AoAC score was higher in those who died than in those who survived, although there was no significant difference in the change in AoAC between the two groups.

There are several important findings in this study. First, CTR increased over time in the HD patients, and the change in CTR was greater in those who died. An increase in CTR on chest radiography has been reported to indicate cardiomegaly, which is associated with cardiac hypertrophy and volume overload. LVH is common in patients with CKD, and starts early in the disease [12]. Foley et al. reported that progressive concentric LVH occurred in 596 incident HD patients without symptomatic cardiac diseases after 2 years of follow-up, and that left ventricular (LV) mass increased in 326 (62.6%) patients and LV volume increased in 258 (49.2%) patients [13]. In addition, several studies have reported associations between LVH and cardiovascular events and mortality in dialysis patients [3,14]. The increase in LV mass in ESRD patients is caused by increases in the LV end-diastolic diameter and LV wall thickness, which is often combined with the features of eccentric and concentric hypertrophy [15]. The mechanisms underlying LVH are complex, and include chronic volume overload, arterial stiffness, elevated systolic blood pressure, anemia, activation of the renin–angiotensin and sympathetic nervous systems, and CKD-related mineral and bone disorders [16]. Although CTR provided a simple and cheap method for detecting patients with LVH and cardiac overload, it could not completely replace echocardiography. Echocardiography could yield important additional information, such as valve function, valve calcification, and regional left ventricular wall motion.

The second important finding of this study is that AoAC significantly increased over time, and that the AoAC score was higher in those who died than in those who survived. Vascular calcification develops early and progresses over time in patients with CKD [17]. Braun et al. were the first to report a marked increase in coronary artery calcium score in HD patients after only 1 year as measured by CT [18]. In addition, Goodman et al. reported that the average calcification score nearly doubled in under 2 years in young patients undergoing dialysis, indicating the rapid progression of coronary artery calcification [19]. These findings suggest that vascular calcification progresses over time after the initiation of HD. However, the degree and rate of vascular calcification varies widely between patients. Bellasi et al. demonstrated that patients with CKD without or with low coronary artery calcification at baseline had minimal progression after 30 months of follow-up [20]. The development of vascular calcification depends on the transdifferentiation of vascular smooth muscle cells to osteoblast-like cells. This can be caused by high calcium and phosphorus burden due to abnormal bone metabolism, oxidative stress, inflammatory cytokines, imbalance in pro-calcific and anti-calcific mediators, cellular apoptosis, the presence of matrix vesicles, uremia, and the use of calcium-containing phosphate binders or calcitriol [4,21].

Analysis of the risk factors affecting the change in CTR and vascular calcification over time showed that an increase in AoAC, older age, female sex, coronary artery disease, and decreased albumin were associated with a greater change in CTR. Age is a non-modifiable cardiovascular risk factor that contributes to arterial stiffness, increased LV afterload, and exacerbated LVH. Hence, an older age may be associated with increased CTR [22]. We also found that the female patients had a higher risk of CTR progression, which is consistent with a study by Cheng et al., who reported that female patients had a 2.5- to 4-fold higher likelihood of developing LVH than male patients after adjusting for potential confounding factors [23]. This may be because a significant difference in cardiac adaptation to pressure overload between males and females has been shown [24]. In addition, we found that a faster decline in albumin was associated with a faster increase in CTR. Chen et al. and Yen et al. reported negative correlations between serum albumin level and CTR in patients without [22] and with diabetes [25] undergoing HD. Furthermore, previous studies have demonstrated relationships among inflammation, malnutrition, and fluid overload in patients undergoing dialysis [26,27,28]. Fluid overload can cause gastrointestinal edema and deficient ingestion, subsequently leading to malnutrition [26]. Inflammatory status can also worsen malnutrition and artherosclerosis [25,28]. Consequently, both fluid overload and artherosclerosis may contribute to the progression of CTR, and partially explain our findings.

Regarding AoAC, older age, cerebrovascular disease, decreased albumin, increased Kt/V, and the use of antiplatelet agents were associated with an increase in AoAC. In addition, we found that a faster decline in albumin was associated with a faster increase in AoAC. The inter-relationships among malnutrition, inflammatory status, and progression of vascular calcification in patients with CKD, which is known as malnutrition–inflammation–atherosclerosis syndrome, have been demonstrated in previous studies [28,29,30]. Another interesting finding in our study is that an increase in ΔKt/V was associated with a higher AoAC, which is consistent with the study by Park et al. [31]. The positive association between high dialysis dose and hemodiafiltration and the severity of vascular calcification may be due to a higher dialysis dose resulting in greater changes in hemodynamics and promoting the loss of fetuin-A, an inhibitor of vascular calcification. Another possible explanation may be that Kt/V is negatively correlated to body weight, and a smaller body size may be associated with malnutrition, chronic inflammation, and atherosclerosis. Another important finding of our study is that the progression of AoAC was positively correlated with the change in CTR. Several studies have shown associations among vascular calcification, arterial stiffness and LVH [32,33]. Vascular calcification results in arterial stiffness, which then increases LV afterload and contributes to LVH. Vascular calcification and LVH have been shown to exacerbate each other and have a longitudinal relationship [33], and the synergistic interaction between vascular calcification and LVH has also been shown to increase the risk of mortality and cardiovascular events [33].

Another major finding of the present study is that the changes in CTR and AoAC over time were more severe in the patients who died than in those who survived, but that only the change in CTR was significant. Previous studies have reported associations between higher baseline CTR and vascular calcification with adverse outcomes in patients undergoing dialysis [3,14,34,35,36]. Other studies have also reported that changes in LVH and AoAC during follow-up were independently associated with cardiovascular events in dialysis patients [37,38,39,40]. Several possible mechanisms may explain how the progression of LVH increases the risk of unfavorable outcomes. LVH is associated with myocardial fibrosis and diastolic dysfunction, both of which contribute to the development of heart failure. In addition, LVH decreases coronary perfusion, leading to cardiac ischemia, myocardial infarction, and life-threatening arrhythmias [37]. In addition, the progression of vascular calcification is associated with arterial stiffness, which then leads to LVH and diastolic heart failure, and ultimately compromised coronary perfusion [36]. However, in our study, there was no significant difference in progression of AoAC between those who died and survived. We hypothesize that one possible mechanism is different types of vascular calcification. AoAC cannot be completely differentiated from intimal and medial vascular calcification on chest radiography, and arterial medial calcification is a much stronger risk factor for the development of arterial stiffness, heart failure, and lethal arrhythmias, leading to cardiovascular events and mortality in dialysis patients. On the other hand, AoAC could be associated with other parallel physiopathological factors that lead to an increase in cardiovascular death.

A strength of this study is that we used repeated measurements to evaluate changes in CTR and AoAC over a longitudinal follow-up period, and few previous studies have investigated the prognostic value of longitudinal changes in CTR and AoAC in dialysis patients. However, there are still several limitations. First, the relatively small study population may have reduced the statistical power of the study. Further studies with a larger sample size are needed to verify our findings. Second, measuring AoAC on plain chest radiographs cannot discriminate between intimal or medial layer calcification. The etiology of vascular calcification contributes to the diverse pathogenesis and clinical outcomes, which may affect the therapeutic strategy. Third, evaluating AoAC on plain chest X-ray is less sensitive than CT, although chest radiography is a simple and non-invasive tool. In addition, body size, overlapping structures, and experience of the observer may influence the assessment of AoAC. Despite prior research indicating that AoAC is positively connected with coronary artery calcium score, it is important to note that coronary artery calcium score is a more accurate tool for predicting cardiovascular events and guiding therapy. Fourth, we did not control for some confounders in this observation study, including body size, fluid status, use of vitamin D, calcium-containing or non-calcium-containing phosphate binders, and cinacalcet, which have been reported to affect survival.

In conclusion, we found that CTR and AoAC increased significantly over time in the HD patients in this study, and that the change in CTR was greater in those who died than in those who survived. Our findings indicate that changes in CTR and AoAC as assessed on simple chest radiography may be a useful marker to predict clinical outcomes in HD patients.

## Figures and Tables

**Table 1:** Comparison of baseline characteristics between patients with CTR > 50 or ≤50 and AoAC >3 or ≤3.

Parameters	CTR > 50% (n = 89)	CTR ≤ 50% (n = 171)	*p*	AoAC > 3.0 (n = 113)	AoAC ≤ 3.0 (n = 147)	*p*
Baseline CTR (%)	55.38 (3.87)	45.76 (2.95)	<0.0001	50.97 (5.81)	47.58 (5.03)	<0.0001
Baseline AoAC	4.80 (4.32)	3.34 (3.64)	0.0044	7.74 (2.62)	0.84 (1.18)	<0.0001
Age (years)	61.73 (12.29)	55.06 (11.78)	<0.0001	63.88 (10.73)	52.32 (11.12)	<0.0001
Sex (male vs. female)	30.3	58.5	<0.0001	43.4	53.4	0.1209
Smoking history (%)	16.9	36.3	0.0011	28.3	30.6	0.6880
Diabetes mellitus (%)	48.3	44.4	0.5523	50.4	42.2	0.1848
Hypertension (%)	77.5	76.6	0.8673	77.0	76.9	0.9818
Coronary artery disease (%)	24.7	18.1	0.2107	32.7	10.9	<0.0001
Cerebrovascular disease (%)	6.7	7.6	0.8002	13.3	2.7	0.0012
Duration of hemodialysis (years)	11.07 (6.13)	12.71 (6.37)	0.0467	12.56 (6.91)	11.84 (5.85)	0.3658
Laboratory parameters						
Albumin (g/dL)	3.90 (0.25)	4.02 (0.37)	0.0096	3.92 (0.25)	4.02 (0.38)	0.0200
Total cholesterol (mg/dL)	182.99 (42.95)	176.49 (53.53)	0.3224	182.62 (54.8)	175.71 (46.28)	0.2717
Triglyceride (mg/dL)	158.6 (104.64)	160.13 (157.63)	0.9341	164.35 (157.89)	155.96 (127.98)	0.6367
Fasting glucose (mg/dL)	137.87 (81.62)	115.59 (59.26)	0.0125	132.66 (74.37)	115.95 (62.75)	0.0507
HbA_1c_ (%)	9.97 (1.19)	10.08 (1.16)	0.4652	10.10 (1.17)	9.99 (1.17)	0.4358
Uric acid (mg/dL)	7.64 (1.58)	7.67 (1.63)	0.9138	7.66 (1.81)	7.66 (1.45)	0.9864
Total calcium (mg/dL)	9.43 (0.99)	9.40 (0.89)	0.8355	9.61 (0.85)	9.26 (0.95)	0.0023
Phosphorous (mg/dL)	4.92 (1.21)	4.91 (1.33)	0.9588	4.91 (1.21)	4.91 (1.35)	0.9868
Calcium–phosphorous product (mg^2^/dL^2^)	46.18 (11.52)	46.1 (13.21)	0.9618	47.12 (11.94)	45.37 (13.12)	0.2695
Kt/V (Daugirdas)	1.60 (0.27)	1.54 (0.51)	0.2926	1.57 (0.26)	1.55 (0.54)	0.7439
Ultrafiltration rate	0.04 (0.01)	0.05 (0.07)	0.3874	0.04 (0.01)	0.05 (0.08)	0.4074
Medications						
Antiplatelet agent use (%)	19.1	12.3	0.1396	23.0	8.2	0.0008
ACEI and/or ARB use (%)	19.1	22.8	0.4904	23.9	19.7	0.4179
Statin use (%)	34.8	25.7	0.1243	29.2	28.6	0.9112

Abbreviations: CTR, cardiothoracic ratio; AoAC, aortic arch calcification; HbA_1c_, glycated hemoglobin; ACEI, angiotensin-converting enzyme inhibitor; ARB, angiotensin II receptor blocker.

**Table 2:** Time course of mean change in CTR or AoAC from baseline (0 year) to 7 years.

Parameters	Total, n = 260					
	Unadjusted LS Means (SE)	*p*	Adjusted LS Means (SE; 95% CI)	*p*	Difference of Adjusted LS Means (SE; 95% CI)	*p*
CTR *						
Test first (0 year) (n = 260)	49.05 (0.35; 48.37 to 49.74)	<0.0001	49.04 (0.32; 48.41 to 49.67)	<0.0001		
To 1 year (n = 260)	49.40 (0.36; 48.69 to 50.11)	0.2490	49.33 (0.32; 48.71 to 49.95)	0.3499	0.28 (0.30; −0.31 to 0.88)	0.3499
To 2 years (n = 225)	50.97 (0.40; 50.18 to 51.75)	<0.0001	50.71 (0.36; 50.00 to 51.42)	<0.0001	1.67 (0.34; 1.01 to 2.33)	<0.0001
To 3 years (n = 190)	50.52 (0.43; 49.68 to 51.37)	<0.0001	50.37 (0.39; 49.59 to 51.14)	0.0002	1.32 (0.35; 0.64 to 2.01)	0.0002
To 4 years (n = 161)	48.51 (0.38; 47.75 to 49.26)	0.1412	48.16 (0.37; 47.44 to 48.88)	0.0184	−0.88 (0.37; −1.61 to −0.15)	0.0184
To 5 years (n = 136)	52.54 (0.54; 51.48 to 53.60)	<0.0001	52.20 (0.49; 51.24 to 53.16)	<0.0001	3.16 (0.48; 2.22 to 4.10)	<0.0001
To 6 years (n = 117)	51.00 (0.49; 50.04 to 51.96)	<0.0001	50.36 (0.45; 49.48 to 51.23)	0.0030	1.32 (0.44; 0.45 to 2.18)	0.0030
To 7 years (n = 56)	51.86 (0.61; 50.67 to 53.06)	<0.0001	50.59 (0.51; 49.59 to 51.58)	0.0018	1.54 (0.49; 0.57 to 2.51)	0.0018
Change in CTR from 0 to 7 years	0.35 (0.06; 0.24 to 0.47)	<0.0001	0.24 (0.06; 0.13 to 0.35)	<0.0001		
AoAC ^†^						
Test first (0 year) (n = 260)	3.84 (0.24; 3.36 to 4.32)	<0.0001	4.05 (0.24; 3.59 to 4.52)	0.3722		
To 1 year (n = 260)	3.59 (0.25; 3.11 to 4.08)	0.1123	3.65 (0.23; 3.20 to 4.11)	0.0112	−0.40 (0.16; −0.71 to −0.09)	0.0112
To 2 years (n = 225)	4.08 (0.26; 3.58 to 4.58)	0.1498	3.97 (0.23; 3.53 to 4.41)	0.6045	−0.08 (0.16; −0.40 to 0.24)	0.6045
To 3 years (n = 190)	3.75 (0.27; 3.22 to 4.27)	0.6482	3.56 (0.24; 3.08 to 4.03)	0.0146	−0.50 (0.2; −0.90 to −0.10)	0.0146
To 4 years (n = 161)	3.73 (0.27; 3.21 to 4.26)	0.6588	3.59 (0.25; 3.10 to 4.09)	0.0673	−0.46 (0.25; −0.95 to 0.03)	0.0673
To 5 years (n = 136)	4.01 (0.28; 3.47 to 4.55)	0.4540	3.73 (0.24; 3.25 to 4.21)	0.1899	−0.32 (0.25; −0.80 to 0.16)	0.1899
To 6 years (n = 117)	4.81 (0.33; 4.16 to 5.45)	0.0010	4.33 (0.30; 3.74 to 4.92)	0.3759	0.27 (0.31; −0.33 to 0.88)	0.3759
To 7 years (n = 56)	6.73 (0.42; 5.90 to 7.56)	<0.0001	6.08 (0.39; 5.31 to 6.86)	<0.0001	2.03 (0.38; 1.29 to 2.77)	<0.0001
Change in AoAC from 0 to 7 years	0.19 (0.04; 0.12 to 0.27)	<0.0001	0.08 (0.04; 0.002 to 0.16)	0.0441		

Abbreviations: CTR, cardiothoracic ratio; AoAC, aortic arch calcification; LS means, least-squares means; SE, standard error; CI, confidence intervals. * Change in mean differences was adjusted for AoAC, age, sex, smoking history, diabetes mellitus, hypertension, coronary artery disease, cerebrovascular disease, duration of HD, albumin, total cholesterol, triglyceride, fasting glucose, HbA_1c_, uric acid, calcium–phosphorous product, Kt/V, ultrafiltration rate, and medications use. ^†^ Change in mean differences was adjusted for CTR, age, sex, smoking history, diabetes mellitus, hypertension, coronary artery disease, cerebrovascular disease, duration of HD, albumin, total cholesterol, triglyceride, fasting glucose, HbA_1c_, uric acid, calcium–phosphorous product, Kt/V, ultrafiltration rate, and medications use.

**Table 3:** Main effects of the variables on CTR or AoAC change over year in hemodialysis patients.

Parameters	Total, n = 260 (CTR Change)		Total, n = 260 (AoAC Change)	
	Adjusted LS Means (SE; 95% CI) *	*p*	Adjusted LS Means (SE; 95% CI) ^†^	*p*
CTR or AoAC change	0.24 (0.06;0.13 to 0.35)	<0.0001	0.08 (0.04; 0.002 to 0.16)	0.0441
CTR (per 1%)	-	-	0.03 (0.02; −0.001 to 0.06)	0.0571
AoAC (per 1 score)	0.11 (0.05; 0.02 to 0.20)	0.0183	-	-
Age > 60 years old	1.55 (0.50; 0.57 to 2.52)	0.0019	1.13 (0.29; 0.56 to 1.71)	0.0001
Sex (male vs. female)	−3.51 (0.70; −4.87 to −2.14)	<0.0001	0.03 (0.52; −0.98 to 1.04)	0.9588
Smoking history	0.46 (0.70; −0.91 to 1.82)	0.5095	0.32 (0.49; −0.64 to 1.28)	0.5163
Diabetes mellitus	0.17 (0.63; −1.07 to 1.41)	0.7856	0.10 (0.36; −0.61 to 0.81)	0.7840
Hypertension	0.31 (0.65; −0.97 to 1.58)	0.6384	0.70 (0.45; −0.18 to 1.58)	0.1173
Coronary artery disease	1.88 (0.65; 0.61 to 3.16)	0.0038	0.50 (0.49; −0.46 to 1.45)	0.3102
Cerebrovascular disease	−1.47 (1.07; −3.57 to 0.63)	0.1688	1.79 (0.68; 0.45 to 3.13)	0.0089
Duration of hemodialysis	−0.06 (0.05; −0.16 to 0.04)	0.2267	0.06 (0.04; −0.01 to 0.13)	0.1052
Laboratory parameters				
Albumin (per 1 g/dL)	−1.86 (0.54; −2.92 to −0.80)	0.0006	−1.17 (0.28; −1.71 to −0.63)	<0.0001
Total cholesterol (per 1 mg/dL)	−0.006 (0.004; −0.014 to 0.014)	0.1060	0.03 (0.002; −0.001 to 0.007)	0.1287
Triglyceride (Ln per 1 mg/dL)	−0.002 (0.001; −0.004 to 0.001)	0.1546	−0.001 (0.000; −0.002 to 0.000)	0.0534
Fasting glucose (Ln per 1 mg/dL)	−0.002 (0.003; −0.007 to 0.003)	0.5176	−0.001 (0.002; −0.004 to 0.003)	0.7285
HbA_1c_ (per 1%)	−0.09 (0.12; −0.33 to 0.16)	0.4879	0.02 (0.07; −0.13 to 0.16)	0.8077
Uric acid (per 1 mg/dL)	−0.05 (0.10; −0.24 to 0.14)	0.6186	−0.04 (0.06; −0.16 to 0.08)	0.4907
Calcium–phosphorous product (per 1 mg^2^/dL^2^)	−0.002 (0.012; −0.026 to 0.021)	0.8426	0.01 (0.01; −0.005 to 0.02)	0.2471
Kt/V (per 1)	−0.36 (0.75; −1.83 to 1.10)	0.6245	1.60 (0.47; 0.68 to 2.51)	0.0006
Ultrafiltration/weight before dialysis (per 1)	0.81 (5.60; −10.17 to 11.79)	0.8855	−1.96 (2.86; −7.57 to 3.65)	0.4937
Medications				
Antiplatelet agent use	0.69 (0.67; −0.63 to 2.01)	0.3080	1.01 (0.45 ; 0.12 to 1.89)	0.0262
ACEI and/or ARB use	−0.34 (0.52; −1.36 to 0.69)	0.5191	0.19 (0.30; −0.41 to 0.78)	0.5377
Statin use	0.18 (0.47; −0.74 to 1.1)	0.6986	0.11 (0.31; −0.50 to 0.72)	0.7249

Abbreviations as Table 2: Data set performs repeated measures for the change in mean differences by using the generalized model. * Change in mean differences was adjusted for AoAC, age > 60 years old, sex, smoking history, diabetes mellitus, hypertension, coronary artery disease, cerebrovascular disease, duration of HD, albumin, total cholesterol, triglyceride, fasting glucose, HbA_1c_, uric acid, calcium–phosphorous product, Kt/V, ultrafiltration rate, and medications use. ^†^ Change in mean differences was adjusted for CTR, age > 60 years old, sex, smoking history, diabetes mellitus, hypertension, coronary artery disease, cerebrovascular disease, duration of HD, albumin, total cholesterol, triglyceride, fasting glucose, HbA_1c_, uric acid, calcium–phosphorous product, Kt/V, ultrafiltration rate, and medications use.

**Table 4:** Comparison of baseline characteristics between patients with or without death.

Parameters	Death (n = 136)	Non-Death (n = 124)	*p*
Baseline CTR (%)	50.43 (0.47)	47.54 (0.49)	<0.0001
Baseline AoAC	5.1 (0.32)	2.45 (0.33)	<0.0001
Age (years)	62.69 (0.94)	51.48 (0.99)	<0.0001
Sex (male vs. female)	52.2	45.2	0.2568
Smoking history (%)	36.0	22.6	0.0185
Diabetes mellitus (%)	58.8	31.5	<0.0001
Hypertension (%)	77.9	75.8	0.6830
Coronary artery disease (%)	27.9	12.1	0.0020
Cerebrovascular disease (%)	12.5	1.6	0.0043
Duration of hemodialysis (years)	9.55 (0.49)	15.01 (0.51)	<0.0001
Laboratory parameters			
Albumin (g/dL)	3.92 (0.03)	4.04 (0.03)	0.0035
Total cholesterol (mg/dL)	174.21 (4.28)	183.65 (4.48)	0.1276
Triglyceride (mg/dL)	162.50 (12.11)	156.43 (12.68)	0.7291
Fasting glucose (mg/dL)	136.96 (5.73)	108.42 (6.03)	0.0006
HbA_1c_ (%)	10.11 (0.10)	9.96 (0.10)	0.2861
Uric acid (mg/dL)	7.60 (0.14)	7.72 (0.14)	0.5545
Total calcium (mg/dL)	9.41 (0.08)	9.42 (0.08)	0.9497
Phosphorous (mg/dL)	4.93 (0.11)	4.89 (0.12)	0.8059
Calcium-phosphorous product (mg^2^/dL^2^)	46.42 (1.08)	45.81 (1.13)	0.6964
Kt/V (Daugirdas)	1.56 (0.04)	1.57 (0.04)	0.9185
Ultrafiltration rate	0.05 (0.08)	0.04 (0.01)	0.5051
Medications			
Antiplatelet agent use (%)	21.3	7.3	0.0021
ACEI and/or ARB use (%)	23.5	19.4	0.4140
Statin use (%)	27.2	30.6	0.5408

Abbreviations as Table 1.

**Table 5:** Time course of mean change in CTR or AoAC from baseline (0 year) to 7 years in the death and non-death groups.

Parameters	Death, n = 136		Non-Death, n = 124		Difference of LS Means (SE; 95% CI)		Adjusted Difference of LS Means (SE; 95% CI) *	
	LS Means (SE)	*p*	LS Means (SE)	*p*		*p*		*p*
CTR								
Test first (0 year)	50.43 (0.47)		47.54(0.48)		2.90 (0.67; 0.96 to 4.83)	0.0003	2.17 (0.72; 0.11 to 4.24)	0.0323
To 1 year	50.96 (0.50)	0.2320	47.70 (0.48)	0.7058	3.26 (0.69; 1.27 to 5.26)	<0.0001	2.35 (0.70; 0.33 to 4.37)	0.0113
To 2 years	52.59 (0.60)	<0.0001	49.22 (0.49)	<0.0001	3.37 (0.77; 1.15 to 5.58)	0.0002	2.35 (0.80; 0.04 to 4.65)	0.0434
To 3 years	52.67 (0.64)	<0.0001	48.35 (0.52)	0.0750	4.34 (0.83; 1.98 to 6.71)	<0.0001	3.36 (0.84; 0.95 to 5.77)	0.0010
To 4 years	50.10 (0.61)	0.5904	46.82 (0.46)	0.1084	3.31 (0.76; 1.11 to 5.51)	0.0002	2.13 (0.83; −0.26 to 4.51)	0.1168
To 5 years	55.79 (0.93)	<0.0001	49.74 (0.57)	<0.0001	6.08 (1.10; 2.96 to 9.20)	<0.0001	4.96 (1.03; 2.04 to 7.89)	<0.0001
To 6 years	53.46 (0.97)	0.0013	48.85 (0.50)	0.0038	4.64 (1.09; 1.50 to 7.77)	0.0003	3.44 (1.04; 0.46 to 6.42)	0.0127
To 7 years	55.46 (1.25)	<0.0001	49.34 (0.63)	0.0009	6.14 (1.40; 2.22 to 10.07)	0.0002	3.94 (1.15; 0.72 to 7.16)	0.0071
Change in CTR from 0 to 7 years	0.60 (0.11)	<0.0001	0.21 (0.06)	0.0002	0.39 (0.13; 0.15 to 0.64)	0.0018	0.30 (0.12; 0.06 to 0.53)	0.0125
AoAC								
Test first (0 year)	5.10 (0.33)		2.45 (0.31)		2.65 (0.46; 1.35 to 3.96)	<0.0001	2.52 (0.49; 1.12 to 3.92)	<0.0001
To 1 year	4.76 (0.35)	0.1648	2.31 (0.31)	0.4362	2.44 (0.47; 1.11 to 3.77)	<0.0001	2.25 (0.50; 0.82 to 3.69)	0.0001
To 2 years	5.57 (0.35)	0.0958	2.49 (0.31)	0.8485	3.08 (0.47; 1.74 to 4.43)	<0.0001	2.80 (0.48; 1.41 to 4.18)	<0.0001
To 3 years	5.22 (0.41)	0.7222	2.21 (0.30)	0.2937	3.01 (0.51; 1.56 to 4.46)	<0.0001	2.79 (0.55; 1.21 to 4.37)	<0.0001
To 4 years	5.42 (0.42)	0.4064	2.04 (0.28)	0.1416	3.38 (0.51; 1.93 to 4.84)	<0.0001	3.18 (0.56; 1.58 to 4.77)	<0.0001
To 5 years	5.64 (0.41)	0.1644	2.41 (0.33)	0.8753	3.23 (0.53; 1.73 to 4.73)	<0.0001	3.00 (0.55; 1.43 to 4.58)	<0.0001
To 6 years	5.90 (0.50)	0.0782	3.52 (0.41)	0.0046	2.38 (0.65; 0.59 to 4.18)	0.0027	2.09 (0.65; 0.26 to 3.92)	0.0156
To 7 years	9.34 (0.63)	<0.0001	4.81 (0.50)	<0.0001	4.53 (0.80; 2.36 to 6.71)	<0.0001	4.07 (0.80; 1.89 to 6.25)	<0.0001
Change in AoAC from 0 to 7 years	0.23 (0.06)	0.0002	0.18 (0.05)	0.0002	0.05 (0.08; −0.10 to 0.21)	0.5006	0.02 (0.08; −0.14 to 0.18)	0.8035
Ln(AoAC)								
Test first (0 year)	1.34 (0.08)		0.68 (0.08)		0.67 (0.11; 0.36 to 0.97)	<0.0001	0.62 (0.11; 0.30 to 0.94)	<0.0001
To 1 year	1.25 (0.08)	0.1191	0.61 (0.08)	0.1540	0.64 (0.11; 0.33 to 0.95)	<0.0001	0.58 (0.12; 0.25 to 0.91)	<0.0001
To 2 years	1.46 (0.07)	0.0618	0.67 (0.08)	0.9636	0.79 (0.11; 0.48 to 1.09)	<0.0001	0.71 (0.11; 0.39 to 1.03)	<0.0001
To 3 years	1.33 (0.09)	0.8896	0.62 (0.08)	0.3525	0.71 (0.12; 0.38 to 1.05)	<0.0001	0.65 (0.13; 0.29 to 1.01)	<0.0001
To 4 years	1.41 (0.09)	0.4361	0.58 (0.07)	0.2171	0.82 (0.12; 0.49 to 1.15)	<0.0001	0.76 (0.13; 0.40 to 1.12)	<0.0001
To 5 years	1.49 (0.09)	0.0814	0.67 (0.08)	0.9759	0.81 (0.12; 0.47 to 1.16)	<0.0001	0.75 (0.13; 0.38 to 1.12)	<0.0001
To 6 years	1.50 (0.10)	0.0757	0.93 (0.10)	0.0046	0.58 (0.14; 0.19 to 0.96)	0.0003	0.50 (0.14; 0.11 to 0.89)	0.0038
To 7 years	2.10 (0.12)	<0.0001	1.25 (0.11)	<0.0001	0.85 (0.16; 0.41 to 1.29)	<0.0001	0.73 (0.16; 0.29 to 1.17)	<0.0001
Change in AoAC from 0 to 7 years	0.05 (0.01)	0.0005	0.05 (0.01)	0.0001	0.00 (0.02; −0.04 to 0.04)	0.9888	−0.01 (0.02; −0.04 to 0.03)	0.7371

Abbreviations as Table 2. * Change in mean differences was adjusted for age > 60 years old, smoking history, diabetes mellitus, coronary artery disease, cerebrovascular disease, duration of HD, albumin, fasting glucose, and antiplatelet agent use. The natural logarithm (Ln) of a number is its logarithm to the base of the mathematical constant e.

**Table 6:** Time course of mean change in CTR or AoAC from baseline (0 year) to 7 years in cardiovascular death and non-death groups.

Parameters	Cardiovascular Death, n = 72		Non-Death, n = 124		Difference of LS Means (SE; 95% CI)		Adjusted Difference of LS Means (SE; 95% CI) *	
	LS Means (SE)	*p*	LS Means (SE)	*p*		*p*		*p*
CTR								
Test first (0 year)	50.98 (0.62)		47.54 (0.48)		3.44 (0.79; 1.17 to 5.71)	0.0002	2.59 (0.79; 0.30 to 4.88)	0.0150
To 1 year	51.07 (0.66)	0.8745	47.70 (0.48)	0.7058	3.37 (0.81; 1.02 to5.72)	0.0005	2.44 (0.81; 0.10 to 4.77)	0.0343
To 2 years	52.65 (0.80)	0.0182	49.22 (0.49)	<0.0001	3.43 (0.94; 0.73 to 6.13)	0.0035	2.52 (0.92; −0.13 to 5.16)	0.0742
To 3 years	53.25 (0.96)	0.0014	48.35 (0.52)	0.075	4.94 (1.09; 1.80 to 8.07)	<0.0001	4.02 (1.06; 0.98 to 7.07)	0.0021
To 4 years	49.75 (0.79)	0.1446	46.82 (0.46)	0.1084	2.98 (0.92; 0.31 to 5.64)	0.0171	1.93 (0.97; −0.85 to 4.71)	0.4058
To 5 years	54.35 (1.20)	0.0037	49.74 (0.57)	<0.0001	4.67 (1.33; 0.86 to 8.48)	0.0062	3.53 (1.27; −0.11 to 7.16)	0.0633
To 6 years	54.13 (1.13)	0.0010	48.85 (0.50)	0.0038	5.33 (1.23; 1.78 to 8.88)	0.0002	4.01 (1.22; 0.49 to 7.52)	0.0143
To 7 years	55.49 (1.28)	0.0002	49.34 (0.63)	0.0009	6.21 (1.43; 2.18 to 10.24)	0.0002	4.92 (1.37; 1.03 to 8.81)	0.0044
Change in CTR from 0 to 7 years	0.50 (0.14)	0.0003	0.21 (0.06)	0.0002	0.30 (0.15; 0.00 to 0.59)	0.0467	0.24 (0.14; −0.04 to 0.52)	0.0917
AoAC								
Test first (0 year)	4.86 (0.44)		2.45 (0.31)		2.41 (0.54; 0.85 to 3.97)	0.0001	2.06 (0.57; 0.44 to 3.68)	0.0037
To 1 year	4.25 (0.45)	0.0904	2.31 (0.31)	0.4362	1.94 (0.54; 0.38 to 3.5)	0.0051	1.52 (0.57; −0.11 to 3.15)	0.0862
To 2 years	5.23 (0.47)	0.356	2.49 (0.31)	0.8485	2.75 (0.56; 1.12 to 4.37)	<0.0001	2.21 (0.53; 0.67 to 3.74)	0.0005
To 3 years	4.24 (0.49)	0.1938	2.21 (0.30)	0.2937	2.04 (0.58; 0.38 to 3.7)	0.0055	1.58 (0.61; −0.17 to 3.32)	0.1092
To 4 years	5.07 (0.54)	0.6863	2.04 (0.28)	0.1416	3.03 (0.61; 1.27 to 4.79)	<0.0001	2.61 (0.66; 0.72 to 4.50)	0.0011
To 5 years	5.22 (0.52)	0.4856	2.41 (0.33)	0.8753	2.82 (0.62; 1.05 to 4.6)	<0.0001	2.38 (0.62; 0.62 to 4.14)	0.0016
To 6 years	4.92 (0.61)	0.9134	3.52 (0.41)	0.0046	1.41 (0.74; −0.67 to 3.48)	0.3790	0.92 (0.70; −1.08 to 2.91)	0.8577
To 7 years	9.00 (0.74)	<0.0001	4.81 (0.50)	<0.0001	4.17 (0.89; 1.71 to 6.62)	<0.0001	3.87 (0.85; 1.52 to 6.22)	<0.0001
Change in AoAC from 0 to 7 years	0.17 (0.07)	0.0185	0.18 (0.05)	0.0002	−0.01 (0.09; −0.18 to 0.16)	0.9103	−0.03(0.09; −0.20 to 0.14)	0.7461
Ln (AoAC)								
Test first (0 year)	1.29 (0.11)		0.68 (0.08)		0.62 (0.13; 0.25 to 0.99)	<0.0001	0.53 (0.14; 0.14 to 0.93)	0.0015
To 1 year	1.14 (0.11)	0.0886	0.61 (0.08)	0.1540	0.53 (0.13; 0.16 to 0.91)	0.0007	0.43 (0.14; 0.04 to 0.83)	0.0223
To 2 years	1.40 (0.11)	0.2148	0.67 (0.08)	0.9636	0.73 (0.13; 0.36 to 1.09)	<0.0001	0.61 (0.13; 0.23 to 1.00)	<0.0001
To 3 years	1.17 (0.12)	0.2589	0.62 (0.08)	0.3525	0.55 (0.14; 0.14 to 0.95)	0.0015	0.44 (0.15; −0.00 to 0.88)	0.0511
To 4 years	1.38 (0.12)	0.4160	0.58 (0.07)	0.2171	0.79 (0.14; 0.40 to 1.19)	<0.0001	0.70 (0.15; 0.27 to 1.13)	<0.0001
To 5 years	1.44 (0.12)	0.1972	0.67 (0.08)	0.9759	0.77 (0.14; 0.35 to 1.18)	<0.0001	0.66 (0.15; 0.24 to 1.09)	0.0001
To 6 years	1.32 (0.13)	0.8399	0.93 (0.10)	0.0046	0.39 (0.16; −0.07 to 0.85)	0.1500	0.28 (0.16; −0.17 to 0.74)	0.5249
To 7 years	1.99 (0.12)	<0.0001	1.25 (0.11)	<0.0001	0.74 (0.16; 0.29 to 1.19)	<0.0001	0.67 (0.16; 0.22 to 1.12)	0.0004
Change in AoAC from 0 to 7 years	0.04 (0.02)	0.0147	0.05 (0.01)	0.0001	−0.01 (0.02; −0.05 to 0.03)	0.6912	−0.01 (0.02; −0.05 to 0.03)	0.6034

Abbreviations as Table 2: * Change in mean differences was adjusted for age > 60 years old, smoking history, diabetes mellitus, coronary artery disease, cerebrovascular disease, duration of HD, albumin, fasting glucose, and antiplatelet agent use. The natural logarithm (Ln) of a number is its logarithm to the base of the mathematical constant e.

**Table 7:** Association between baseline CTR and AoAC with overall mortality.

	Death vs. Non-Death		Death vs. Non-Death for CTR		Death vs. Non-Death for CTR AoAC	
Parameters	Crude OR (95% CI)	*p*	Adjusted OR (95% CI) *	*p*	Adjusted OR (95% CI) *	*p*
Baseline CTR (per 1%)	1.10 (1.05–1.16)	<0.0001	1.09 (1.02–1.17)	0.0133		
Baseline AoAC (per 1 score)	1.21 (1.13–1.30)	<0.0001			1.23 (1.11–1.37)	<0.0001
Age > 60 years old	7.32 (4.14–12.95)	<0.0001	4.05 (1.94–8.45)	0.0002	2.47 (1.11–5.50)	0.0262
Sex (male vs. female)	1.33 (0.81–2.16)	0.2568	2.16 (0.78–6.00)	0.1379	1.62 (0.58–4.55)	0.3560
Smoking history	1.93 (1.12–3.34)	0.0185	1.13 (0.45–2.87)	0.7905	1.07 (0.41–2.78)	0.8933
Diabetes mellitus	3.11 (1.87–5.19)	<0.0001	1.70 (0.76–3.81)	0.1950	1.60 (0.71–3.64)	0.2575
Hypertension	1.13 (0.63–2.01)	0.6830	0.28 (0.11–0.68)	0.0053	0.25 (0.10–0.63)	0.0033
Coronary artery disease	8.71 (1.97–38.54)	0.0043	4.43 (0.81–24.21)	0.0862	2.59 (0.48–14.06)	0.2697
Cerebrovascular disease	2.82 (1.46–5.44)	0.0020	2.64 (1.03–6.72)	0.0423	2.46 (0.94–6.45)	0.0668
Duration of hemodialysis	0.84 (0.80–0.89)	<0.0001	0.87 (0.81–0.93)	0.0001	0.83 (0.77–0.90)	<0.0001
Laboratory parameters						
Albumin (per 1 g/dL)	0.27 (0.11–0.67)	0.0050	0.62 (0.17–2.28)	0.4695	0.60 (0.16–2.28)	0.4515
Total cholesterol (per 1 mg/dL)	1.00 (0.99–1.00)	0.1409	1.00 (0.99–1.00)	0.4268	0.99 (0.98–1.00)	0.1253
Triglycerides (Ln per 1 mg/dL)	1.00 (0.99–1.00)	0.7299	1.00 (0.99–1.00)	0.7710	1.00 (0.99–1.00)	0.9583
Fasting glucose (Ln per 1 mg/dL)	1.01 (1.00–1.01)	0.0014	1.00 (0.99–1.01)	0.4585	1.00 (0.99–1.01)	0.2876
HbA_1c_ (per 1%)	1.12 (0.91–1.38)	0.2894	1.15 (0.84–1.57)	0.3930	1.07 (0.78–1.47)	0.6706
Uric acid (per 1 mg/dL)	0.96 (0.82–1.11)	0.5553	1.05 (0.84–1.31)	0.6715	1.05 (0.84–1.31)	0.6893
Calcium–phosphorus product (per 1 mg^2^/dL^2^)	1.00 (0.98–1.02)	0.6970	1.01 (0.99–1.04)	0.3622	1.01 (0.98–1.04)	0.4255
Kt/V (per 1)	0.97 (0.56–1.69)	0.9185	2.21 (0.64–7.62)	0.2099	1.92 (0.58–6.32)	0.2842
Ultrafiltration/weight before dialysis (per 1)	0.96 (0.75–1.22)	0.7299	1.12 (0.79–1.60)	0.5264	1.06 (0.74–1.52)	0.7548
Medications						
Antiplatelet agent use	3.46 (1.57–7.65)	0.0021	1.10 (0.38–3.16)	0.8566	0.90 (0.32–2.55)	0.8421
ACEI and/or ARB use	1.28 (0.71–2.33)	0.4140	1.96 (0.86–4.46)	0.1072	1.47 (0.63–3.41)	0.3715
Statin use	0.85 (0.49–1.45)	0.5408	0.63 (0.29–1.38)	0.2510	0.76 (0.34–1.68)	0.4912

* The Hosmer–Lemeshow goodness-of-fit test indicated that the model was well calibrated (*p =* 0.5420 for CTR and 0.3987 for AoAC).

**Table 8:** Association of ΔCTR and ΔAoAC with overall mortality.

	Death vs. Non-Death		Death vs. Non-Death for ΔCTR		Death vs. Non-Death for ΔAoAC	
Parameters	Crude OR (95% CI)	*p*	Adjusted OR (95% CI) *	*p*	Adjusted OR (95% CI) *	*p*
ΔCTR (per 1%)	1.07 (1.02–1.12)	0.0083	1.13 (1.06–1.21)	0.0003		
ΔAoAC (per 1 score)	0.97 (0.89–1.04)	0.3599			0.97 (0.88–1.08)	0.6084
Age > 60 years old	7.32 (4.14–12.95)	<0.0001	5.10 (2.41–10.78)	<0.0001	4.85 (2.35–10.00)	<0.0001
Sex (male vs. female)	1.33 (0.81–2.16)	0.2568	1.38 (0.51–3.74)	0.5249	1.63 (0.62–4.26)	0.3185
Smoking history	1.93 (1.12–3.34)	0.0185	1.32 (0.52–3.34)	0.5562	1.23 (0.5–3.02)	0.6559
Diabetes mellitus	3.11 (1.87–5.19)	<0.0001	1.41 (0.63–3.13)	0.4041	1.41 (0.65–3.07)	0.3839
Hypertension	1.13 (0.63–2.01)	0.6830	0.26 (0.10–0.66)	0.0047	0.31 (0.13–0.76)	0.0108
Coronary artery disease	8.71 (1.97–38.54)	0.0043	3.31 (0.60–18.22)	0.1689	3.98 (0.74–21.4)	0.1071
Cerebrovascular disease	2.82 (1.46–5.44)	0.0020	3.13 (1.15–8.51)	0.0253	2.93 (1.15–7.48)	0.0245
Duration of hemodialysis	0.84 (0.80–0.89)	<0.0001	0.86 (0.80–0.92)	<0.0001	0.87 (0.81–0.93)	<0.0001
Laboratory parameters						
Albumin (per 1 g/dL)	0.27 (0.11–0.67)	0.0050	0.33 (0.08–1.33)	0.1197	0.50 (0.14–1.84)	0.2971
Total cholesterol (per 1 mg/dL)	1.00 (0.99–1.00)	0.1409	0.99 (0.99–1.00)	0.1585	1.00 (0.99–1.00)	0.4028
Triglycerides (Ln per 1 mg/dL)	1.00 (0.99–1.00)	0.7299	1.00 (0.99–1.00)	0.7201	1.00 (0.99–1.00)	0.6636
Fasting glucose (Ln per 1 mg/dL)	1.01 (1.00–1.01)	0.0014	1.01 (0.99–1.01)	0.1171	1.00 (0.99–1.01)	0.2347
HbA_1c_ (per 1%)	1.12 (0.91–1.38)	0.2894	1.15 (0.85–1.56)	0.3639	1.11 (0.82–1.5)	0.4871
Uric acid (per 1 mg/dL)	0.96 (0.82–1.11)	0.5553	1.05 (0.84–1.32)	0.6745	1.08 (0.87–1.35)	0.4830
Calcium–phosphorus product (per 1 mg^2^/dL^2^)	1.00 (0.98–1.02)	0.6970	1.02 (0.99–1.05)	0.2395	1.02 (0.99–1.04)	0.2856
Kt/V (per 1)	0.97 (0.56–1.69)	0.9185	3.21 (0.83–12.38)	0.0902	2.46 (0.68–8.92)	0.1709
Ultrafiltration/weight before dialysis (per 1)	0.96 (0.75–1.22)	0.7299	1.03 (0.71–1.47)	0.8880	1.10 (0.77–1.56)	0.6045
Medications						
Antiplatelet agent use	3.46 (1.57–7.65)	0.0021	1.15 (0.41–3.24)	0.7922	1.18 (0.42–3.29)	0.7575
ACEI and/or ARB use	1.28 (0.71–2.33)	0.4140	1.63 (0.71–3.75)	0.2466	1.74 (0.78–3.89)	0.1762
Statin use	0.85 (0.49–1.45)	0.5408	0.79 (0.36–1.74)	0.5574	0.69 (0.32–1.48)	0.3395

Adjusted odds ratio (OR) was estimated by a multiple logistic regression model. * The Hosmer–Lemeshow goodness-of-fit test indicated that the model was well calibrated (*p* = 0.9456 for CTR and 0.6419 for AoAC).

## Data Availability

Data may be available upon request to interested researchers. Please send data requests to: Szu-Chia Chen, PhD, MD. Division of Nephrology, Department of Internal Medicine, Kaohsiung Medical University Hospital, Kaohsiung Medical University.
